# MicroRNA-148 as a negative regulator of the common TLR adaptor mediates inflammatory response in teleost fish

**DOI:** 10.1038/s41598-017-04354-9

**Published:** 2017-06-23

**Authors:** Qing Chu, Yunhang Gao, Dekun Bi, Tianjun Xu

**Affiliations:** Laboratory of Fish Biogenetics & Immune Evolution, College of Marine Science, Zhejiang Ocean University, Zhoushan, 316022 China

## Abstract

MicroRNAs are small endogenous noncoding RNAs implicating in the regulation of diverse biological processes, including proliferation, differentiation, cancer, apoptosis, and viral infections. MicroRNAs regulate gene expression by either mRNA degradation or inhibition of protein translation. Although microRNAs have emerged as important controller involved in regulation of inflammatory response, the microRNA-mediated regulatory mechanism remains less clear in teleost. Here, we report that miR-148 targets MyD88 and down-regulates its expression by inhibition protein translation rather than degradation mRNA in miiuy croaker. Additionally, we found that miR-148 was significantly upregulated in miiuy croaker after treated with *Vibro h*arveyi, as well as LPS. Overexpression of miR-148 inhibited LPS-induced inflammatory cytokines production, such as IL-6 and IL-1β, which then avoid excessive inflammation response. miR-148 has also been identified to suppress NF-κB pathway through targeting and repressing MyD88 expression. Taken together, our findings indicate that miR-148 participates in bacteria-induced inflammatory response and act as a negative regulator for MyD88-mediated NF-κB signaling, which may clarify the mechanism of microRNAs for avoiding excessive inflammation in teleost fish.

## Introduction

The innate immune system could be activated via recognition of specific pathogen-associated molecular patterns (PAMPs) by toll-like receptors which sequentially trigger the cascade of inflammatory responses against the invasion of pathogenic microorganisms^1, 2^. Most TLRs, upon recognition of PAMPs in microbial components, bind adaptor proteins (e.g. MyD88) which in turn recruit the interleukin-1 receptor-associated kinase (IRAK) complex, including IRAK1, IRAK2, IRAK4 and IRAKM^3^. Once IRAK1 phosphorylated, IRAKs associate with TNF receptor-associated factor 6 (TRAF6) and lead to the activation of NF-κB and MAPKs signaling^4–6^. The IRAK-TRAF6 complex induces the phosphorylation and degradation of IκB through the IKK complex, and then activates the nuclear translocation of NF-κB that promotes the expression of various inflammatory genes, including IL-1β, IL-6 for defending against invaders^6^. However, excessive TLR signaling activation and overproduction of inflammatory cytokines will disrupt immune homeostasis, further may induce the autoimmune and inflammatory diseases^7^. As such, it is essential to study the mechanisms for the negative regulation of TLR signaling.

MicroRNAs (miRNAs) are a class of highly conserved, regulatory, small noncoding RNAs that bind to the 3′-untranslated regions (3′-UTRs) of target mRNAs and recruit the RNA-induced silencing complex leading to either mRNA degradation or translational suppression, thus effectively silencing their mRNA targets^8, 9^. As reported, as many as 60% of all mRNAs have been predicted to be regulated by miRNAs to some extent^10^. It is therefore that miRNAs serve as important as transcription factors in controlling the protein content of a cell. Given the important roles of TLR-signaling pathway in inflammatory response, more miRNAs have been identified in regulation TLR-signaling pathway at different layers^11^. MiR-132, miR-212, and miR-146a are known to target IRAK4 and then decreasing the production of inflammatory cytokines^12^. Moreover, miRNAs are also reported to negatively regulate the NF-κB pathway, For instance, miR-146a inhibits NF-κB activity by targeting TRAF6^13^. However, the underlying mechanisms of miRNAs in regulation of inflammatory responses against varied pathogens or stimuli remain poorly understood for teleost fish. Here, we found that the expression of miR-148 was significantly up-regulated following infecting with *Vibro harveyi* in miiuy croaker (*Miichthys miiuy*). Further investigation revealed that miR-148 inhibits NF-κB activity and inflammatory cytokine productions, including IL-6 and IL-1β by targeting and down-regulating MyD88 in macrophages treated with LPS stimulation. Our studies suggested that miR-148 acts as a negative regulator of MyD88 dependent NF-κB signaling in teleost, which may provide a new insight into the regulatory mechanism of the innate signaling pathway in mammals.

## Results

### Upregulation of miR-148 upon V. harveyi and LPS stimulation

**Figure 1:**
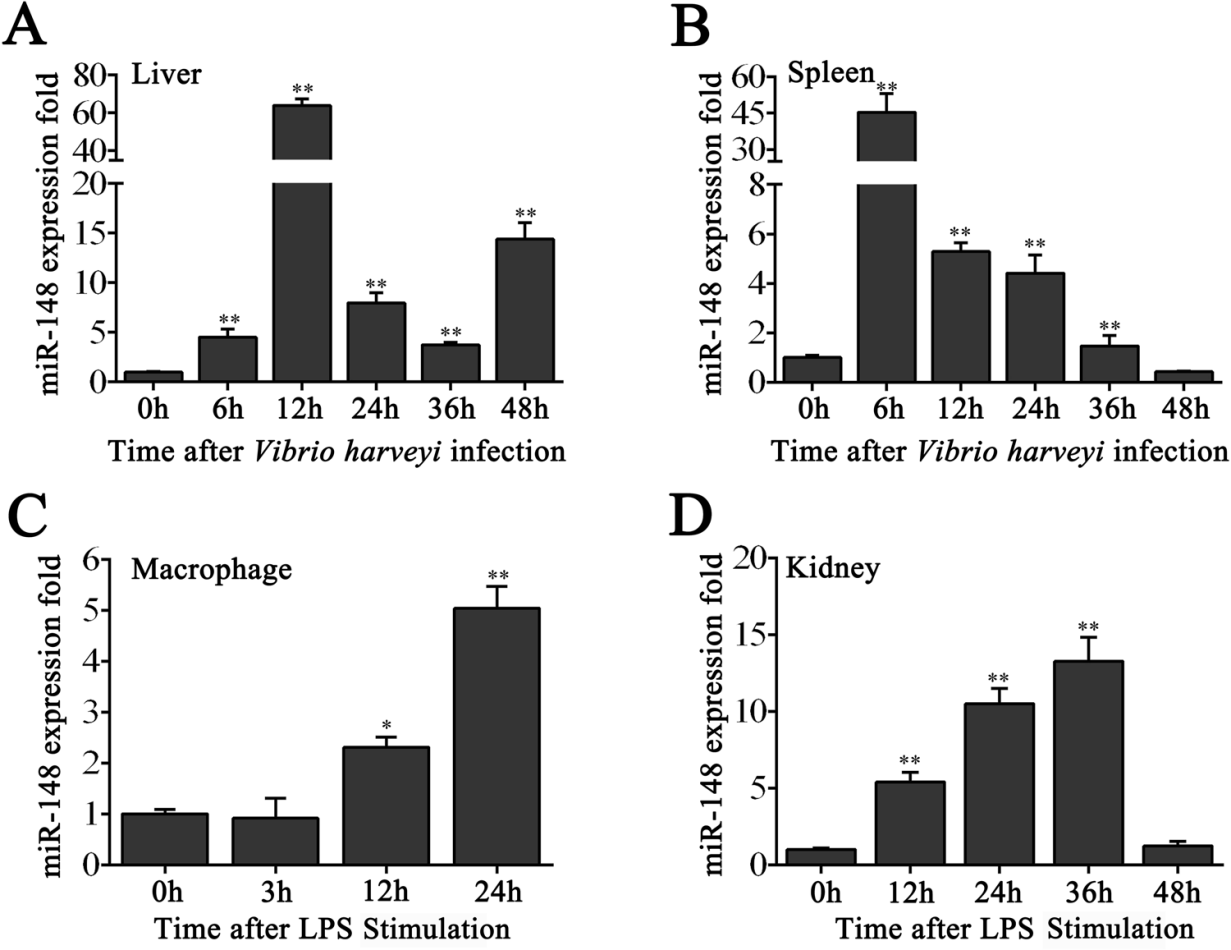
The expression profiles of miR-148. Expression profiles of miR-148 after *V. harveyi* infection in liver (**A**) and spleen (**B**). Expression profiles of miR-148 after LPS stimulation in macrophages (**C**) and kidney (**D**). Results are standardized to 1 in control cells. Data was represented as the mean ± SE from three independent triplicated experiments. **p < 0.01; *p < 0.05 versus the controls.

In order to test the effect of *V. harveyi* infection on the miRNA profile, a small RNAs deep-sequencing analysis of miiuy croaker spleen challenged with *V. harveyi* was performed. Deep-sequencing data indicated that some of miRNAs were differentially expressed upon *V. harveyi* injection (data not shown). Among these miRNAs, miR-148 was shown to be significantly upregulated following stimulation. To better explore the expression profiles of miR-148 upon pathogen stimulation, we investigated the levels of miR-148 in *V. harveyi* infected miiuy croaker liver and spleen samples using qRT-PCR. The results showed that the expression of miR-148 in both liver and spleen samples was rapidly up-regulated and reached a peak at 12 h and 6 h, respectively (Fig. 1A,B).

Additionally, we tried to explore the expression profiles of miR-148 *in vitro*. To this end, miR-148 expression was investigated in LPS exposure macrophages. As shown in Fig. 1C, the expression of miR-148 kept the rising trend in macrophages after LPS stimulation, and reached a peak at 24 h. As macrophages were aseptically obtained from kidney of miiuy croaker, we thus used LPS, the endotoxin of Gram-negative bacteria, to stimulate kidney sample. As shown in Fig. 1D, LPS also exhibited an activating effect on miR-148 expression in kidney sample of miiuy croaker. Taken together, our findings demonstrate that miR-148 expression could be upregulated by *V. harveyi*, as well as LPS, and miR-148 may be involved in the regulation of immune response.

### miR-148 inhibits LPS-induced inflammatory cytokine production

**Figure 2:**
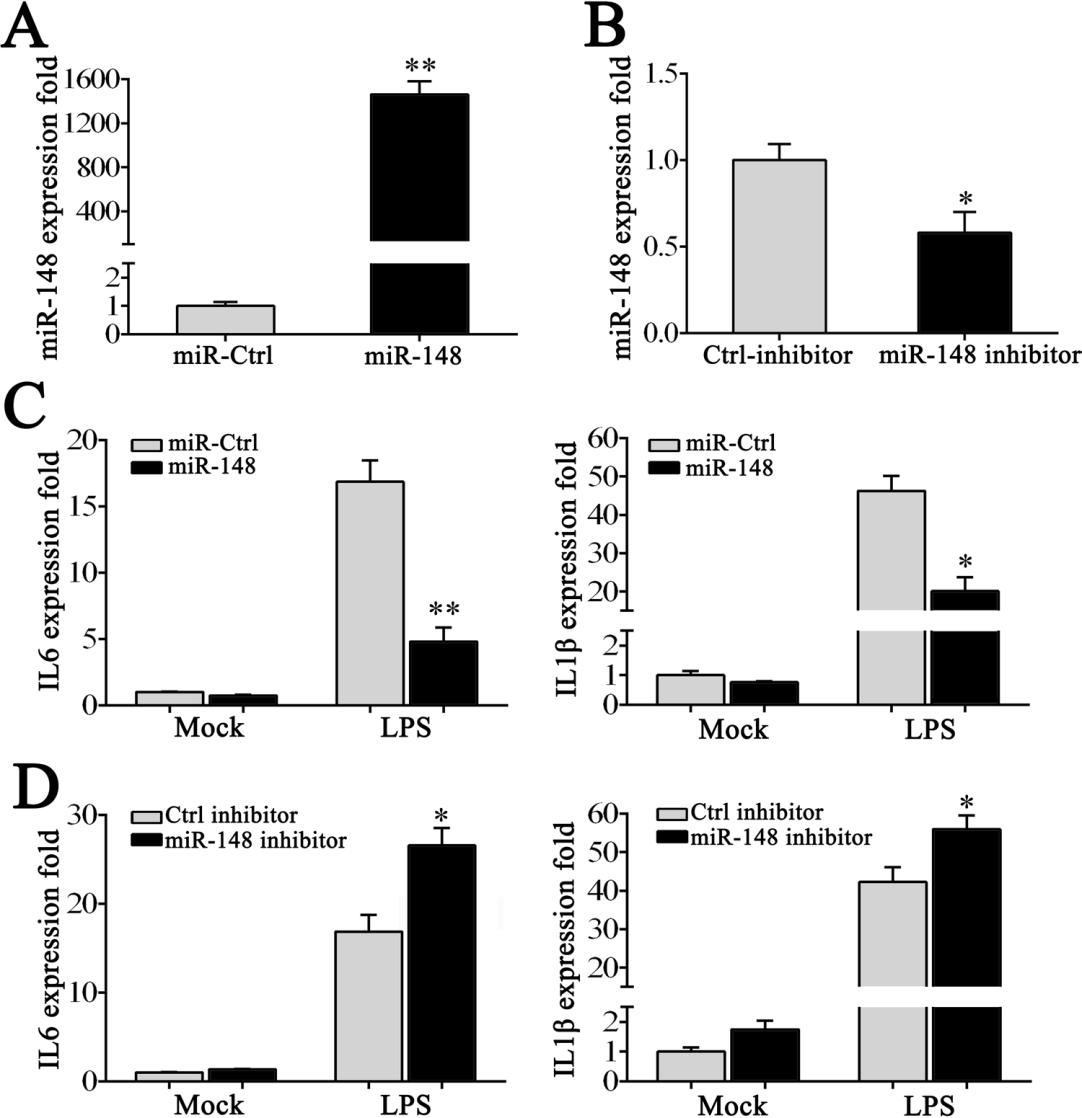
miR-148 suppresses the expression of IL-6 and IL-1β in LPS-treated macrophages. Miiuy croaker macrophages were transfected with miR-Ctrl or miR-148 mim﻿ics (**A**) and Ctrl-inhibitor or miR-148 inhibitor (**B**) within a final concentration of 100 nM. After 24 h post-transfection, miR-148 expression was measured by qRT-PCR. The macrophages were transfected with miR-148 mimics or miR-Ctrl (**C**) and miR-148 inhibitor or Ctrl-inhibitor (**D**). After 24 h, the cells were stimulated with LPS for another 24 h, and the expression levels of IL-6 and IL-1β were analyzed by qRT-PCR. Physiological water served as stimulation control. Data was presented as the means ± SE from three independent triplicated experiments. **p < 0.01; *p < 0.05 versus the controls.

To verify the role of miR-148 in cytokine production, we first transfected with miR-148 mimics into macrophages to overexpress miR-148. 24 h post-transfection, the expression level of miR-148 was measured by qRT-PCR. As shown in Fig. 2A, miR-148 mimics increased miR-148 expression approximately 1400-fold in macrophages. Then, we test the regulation role of miR-148 on the production of inflammatory cytokines in macrophages after LPS stimulation. To this end, miR-148 mimics was transfected into macrophages for 24 h, which were then treated with LPS for another 24 h. Compared to control miRNA, overexpression miR-148 markedly decreased the expression of IL-6, IL-1β in macrophages after LPS stimulation (Fig. 2C).

To further confirm the function of miR-148 in LPS-induced production of inflammatory cytokines, miR-148 inhibitor was transfected into macrophages. As shown in Fig. 2B, transfection of miR-148 inhibitor decreases miR-148 expression in macrophages compared to control inhibitor (Fig. 2B). After 24 h transfection of miR-148 inhibitor into macrophages, we then used LPS to stimulate cells for another 24 h. As shown in Fig. 2D, we observed that both the expression of IL-6 and IL-1β were increased in macrophages after transfection with miR-148 inhibitor compared to control inhibitor. These data demonstrate that miR-148 could suppress the production of certain inflammatory cytokines, including IL-6 and IL-1β, which may play negative role in response to LPS stimulation.

### MyD88 is the target of miR-148

**Figure 3:**
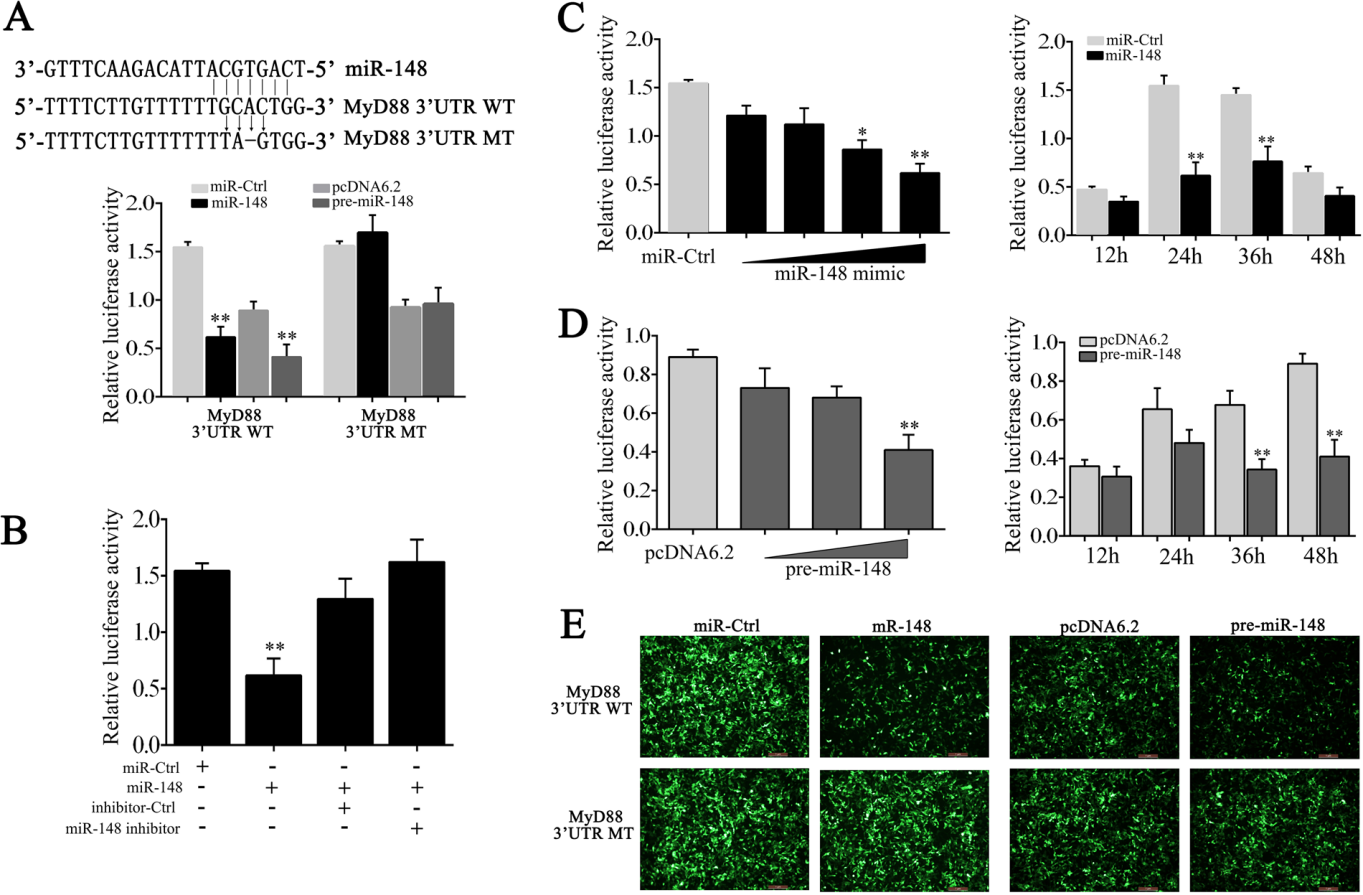
miR-148 targets miiuy croaker MyD88. (**A**) HEK293 cells were cotransfected with the wild type of MyD88-3′UTR (WT) and the mutant type of MyD88-3′UTR (MT), together with miR-148 or miR-Ctrl and pre-miR-148 or its control. After 24 h, luciferase activity was determined and normalized to renilla luciferase activity. (**B**) HEK293 cells were cotransfected with MyD88-3′UTR (WT), together with miR-148 or miR-Ctrl and miR-148 inhibitor or Ctrl-inhibitor. After 24 h, luciferase activity was determined and normalized to renilla luciferase activity. (**C**) A concentration and time gradient experiment were performed for miR-148 mimics. (**D**) A concentration and time gradient experiment were performed for pre-miR-148. (**E**) HEK293 cells were cotransfected with pIZ/EGFP-MyD88-3′UTR or empty vector, together with miR-148 or miR-Ctrl and pre-miR-148 or pcDNA6.2. 48 h post-transfection, the fluorescence intensity was evaluated. Data was presented as the means ± SE from three independent triplicated experiments. **p < 0.01; *p < 0.05 versus the controls.

**Figure 4:**
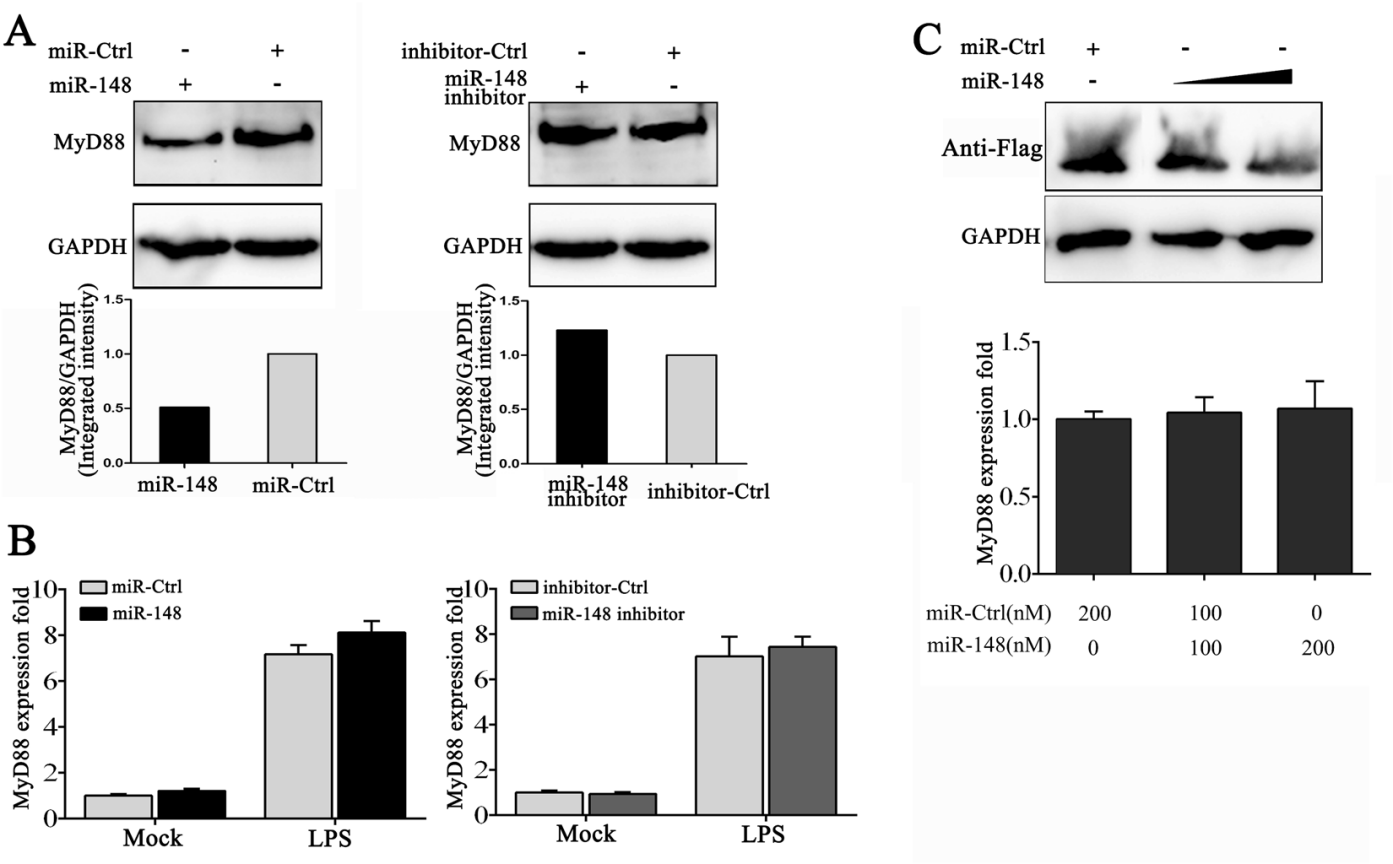
miR-148 inhibits the expression of MyD88. (**A**) The miiuy croaker macrophages were transfected with miR-148 or miR-Ctrl and miR-148 inhibitor or Ctrl-inhibitor for 24 h, which then stimulated with LPS for another 24 h. After 48 h transfection, the protein levels of MyD88 were determined by Western blot. Mock group represents the macrophages without stimulation. Cropped blots are shown for antibodies For un-cropped blots, see Supplemental Figure 2. (**B**) The mRNA levels of MyD88 were also determined by qRT-PCR at 48 h post-transfection. (**C**) HEK293 cells were transfected with MyD88 expression plasmid containing Flag tag, together with miR-148 or miR-Ctrl with concentrate gradient. After 48 h, the anti-Flag Western blot was conducted to detect the protein levels of MyD88. A cropped Flag blot is shown. For un-cropped blots, see Supplemental Figure 2. The mRNA levels of MyD88 were also determined after 48 h transfection. The mRNA expression profiles was normalized to the expression of β-actin and represented as fold induction relative to the expression level in control cells that was set to 1. Data was presented as the means ± SE from three independent triplicated experiments.

To investigate the potential target of miR-148, we used the TargetSan^14^ software to predict miR-148 targets and found putative miR-148 binding sites in the 3′UTR of miiuy croaker MyD88 (Fig. 3A). To verify the prediction, we transfected with miR-148 mimics or control miRNA, together with the reporter vector containing the wild-type or miR-148 seed-binding site mutated 3′UTR sequence of MyD88 into HEK293 cells. 24 h post-transfection, we then performed dual-luciferase reporter assays on HEK293 cells. As shown in Fig. 3A, miR-148 could decrease the luciferase activity by 60.09% compared to control miRNA, while mutant-type led to a complete abrogation of the negative effect. To further verify this result, a concentration and time gradient experiment were performed (Fig. 3C). The significant down-regulation mechanism was further investigated using miR-148 mimics and inhibitor, revealing that inhibition of luciferase activity was attenuated after co-transfection with miR-148 inhibitor (Fig. 4B).

Additionally, given that miRNA processing system is conserved from invertebrates to vertebrates^15^, we then transfected with pre-miR-148 plasmid into HEK293 cells for *in vitro* expression. The results demonstrate that miR-148 could be detected in the transfected cell using qRT-PCR and RT-PCR (Supplemental Fig. 1). Subsequently, pre-miR-148 plasmid was transfected into HEK293 cell, together with wild-type or mutant-type MyD88 3′UTR. As shown in Fig. 1A, pre-miR-148 plasmid was sufficient to luciferase activity compared to control. In contrast, both the control and pre-miR-148 showed no effect on the luciferase activity of mutant-type. The result was further verified within concentration and time gradient experiment (Fig. 3D). Moreover, as shown in Fig. 3E, both miR-148 mimics and pre-miR-148 plasmid could downregulate GFP gene expression when the MyD88-3′UTR was cloned into the pIZ/EGFP vector in HEK293 cells. Collectively, these results indicate that the nucleotide sequence in the 3′UTR sequence of MyD88 is a potential miR-148 targeting site.

### miR-148 regulates MyD88 expression by inhibition protein translation but not degradation mRNA

To further validate miR-148 in regulation of MyD88 expression, the expression of MyD88 was examined in miiuy croaker macrophages treated with miR-148 mimics and inhibitor. As expected, overexpression of miR-148 could suppress MyD88 protein levels, whereas the suppression of MyD88 could be restored in the presence of miR-148 inhibitor (Fig. 4A). We further investigated whether miR-148 acts to affect the stability of MyD88 mRNA. To this end, miiuy croaker macrophages were transfected with miR-148 mimics or inhibitor after treated with LPS. As shown in qRT-PCR (Fig. 4B), either miR-148 mimics or inhibitor could not affect the expression of MyD88 mRNA. Additionally, we also sought to transfect with miR-148 mimics, together with MyD88 expression plasmid into HEK293 cells. To construct MyD88 expression plasmid, the full length CDS region and 3′UTR of miiuy croaker MyD88 gene was amplified by specific primer pairs and cloned into pcDNA3.1 vector with Flag tag. As shown in Fig. 4C, miR-148 decreased MyD88 protein level but not mRNA level. Thus, it can be concluded that miR-148 decreases MyD88 protein expression by repressing its protein translation but does not affect its mRNA stability.

### miR-148 suppresses MyD88-mediated NF-κB pathway

**Figure 5:**
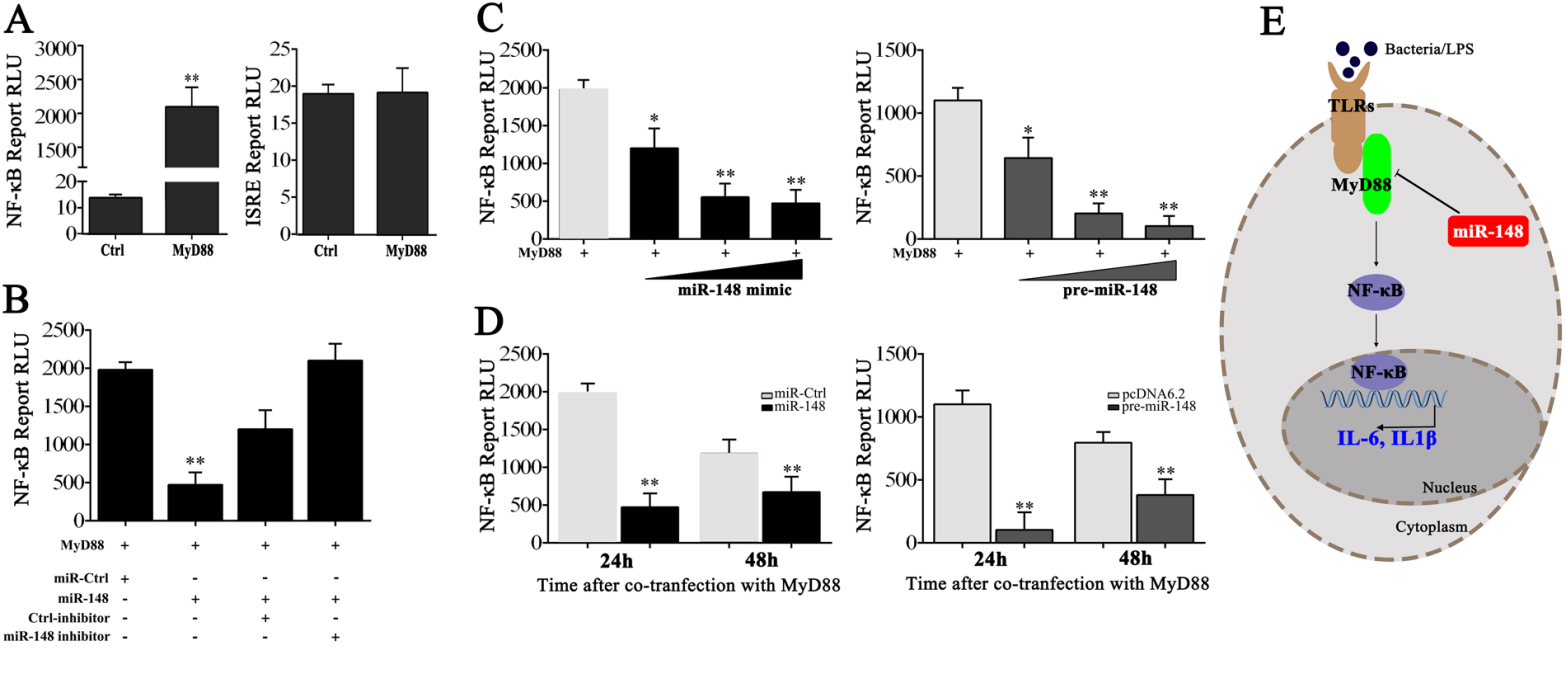
miR-148 suppresses MyD88-mediated NF-κB pathway. (**A**) HEK293 cells were transfected with MyD88 expression plasmid, together with luciferase reporter constructs driven by NF-κB, and ISRE reporter genes. After 24 h, the luciferase activity measured and results were presented relative to the luciferase activity in control cell. (**B**) miR-148 or miR-Ctrl and miR-148 inhibitor or Ctrl-inhibitor was transfected with MyD88 expression plasmid, together with luciferase reporter NF-κB into HEK293 cells, each assay was transfected within equal amount of oligonucleotides. After 24 h, the luciferase activity measured. (**C**,**D**) A concentration and time gradient experiment were performed for miR-148 mimics and pre-miR-148 plasmid. (**E**) A proposed model for miR-148 of inflammatory cytokine secretion via miiuy croaker MyD88. The relative luciferase activity value was achieved against the renilla luciferase control. Data was presented as the means ± SE from three independent triplicated experiments. **p < 0.01; *p < 0.05 versus the controls.

To determine the regulation role of miiuy croaker MyD88, we then constructed MyD88 expression plasmid which containing the full length CDS region and 3′UTR of miiuy croaker MyD88 gene, and then transfected with it into HEK293 cells. As shown in Fig. 5A, MyD88 was sufficient to activate NF-κB reporter gene, whereas it shows no obvious effect on interferon-stimulated response element (ISRE) reporter gene. Given that miR-148 targets and negatively regulates the expression of miiuy croaker MyD88, we then examined whether overexpression of miR-148 inhibits the activation of NF-κB reporter gene. 24 h post-transfection, miR-148 mimics significantly attenuated the activation of NF-κB induced by overexpression of MyD88, whereas the negative effect was abrogated after co-transfected with miR-148 inhibitor (Fig. 5B). To better demonstrate the negative role of miR-148 for NF-κB activation, concentration and time gradient experiments were conducted (Fig. 5C,D). Taken together, a proposed model for miR-148 regulation in miiuy croaker has been performed (Fig. 5E). These data demonstrate that miR-148 negatively regulates NF-κB pathway in a manner that depends on targeting and inhibiting MyD88 expression.

### miR-148 inhibits inflammatory cytokines production through MyD88

**Figure 6:**
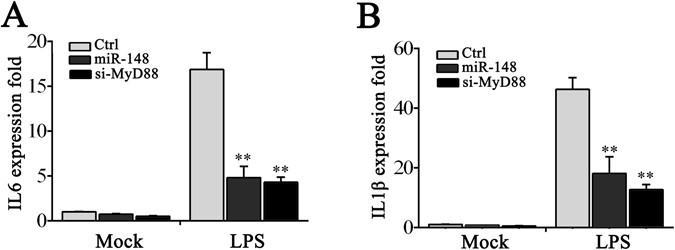
siRNA interference with MyD88 in miiuy croaker macrophages. Miiuy croker macrophages were transfected with control siRNA (si-Ctrl) or siRNA against MyD88 (si-MyD88). After 24 h transfected, miiuy croaker macrophages were then stimulated with LPS for another 24 h and the mRNA expression of IL-6 (**A**) and IL-1β (**B**) were determined, respectively. Mock group represents the macrophages without stimulation. The mRNA expression profile was normalized to the expression of β-actin and represented as fold induction relative to the expression level in control cells that was set to 1. Data was presented as the means ± SE from three independent triplicated experiments. **p < 0.01 versus the controls.

To confirm the contribution of MyD88 on regulation of inflammatory cytokines, the miiuy croaker macrophages were transfected with MyD88-specific small interfering RNA (siRNA), and results showed that the expression levels of MyD88 mRNA and protein were effectively inhibited^16^. Afterwards, we silenced MyD88 and examined the production of inflammatory cytokines in macrophages treated with LPS stimulation. As shown in Fig. 6A and B, knockdown of MyD88 significantly decreased the levels of IL-6, IL-1β mRNA in macrophages after LPS stimulation, which produced effect similar to that of miR-148 overexpression. The results indicate that bacteria induced miR-148 regulates inflammatory cytokines production through inhibition of MyD88, and thus suppressing inflammatory cytokine production.

## Discussion

*Vibrio harveyi*, a marine Gram-negative bacteria, is a serious pathogen for a wide range of marine animals^17^. Recently, the development of the miiuy croaker aquaculture has been hindered by *V. harveyi*, leading to the high mortality. Miiuy croaker, a member of the Sciaenidae family, is an economically important marine fish. Evidences indicate that miiuy croaker has been studied in-depth from transcriptome^18, 19^, whole-genome^20^, to functional genes, which left miiuy croaker to be a new model for studying the immune system in fish. In response to bacterial infection, innate immunity and a series of inflammatory response are the main defense system in fish^21^. However, excessive inflammatory may disrupt immune homeostasis, leading to some diseases and even death. Thus, mechanisms for the negative regulation of immune response are especially important.

Upon recognition of PAMPs, almost all TLRs bind MyD88 leading to the transcription of inflammatory cytokines and then transcriptionally regulate host-cell response to pathogens^22^. MyD88 is a key downstream adaptor for most Toll-like receptors and interleukin-1 receptors^23^. Recently studies demonstrated that the structure of MyD88 is well conserved and its homolog in fish species may function similarly to the mammalian counterparts^24^. Until now, an array of miRNAs has been indicated to regulate MyD88 in mammal, including miR-155, miR-149 and miR-203^25^. In lower vertebrates like fish, few evidences exist to support the direct regulation of MyD88. Recently, we first discover that miR-3570 is responsible for the inflammatory reaction by targeting MyD88 after *Vibrio anguillarum* infection, which suggest the mechanism for miRNA regulation in teleost fish, as well as in mammals^16^. Previous studies indicated that each highly conserved miRNA probably targets several hundred distinct mRNAs, and each gene could be regulated by lots of miRNAs. Thus, in our present paper, we found another new microRNA, miR-148 could also target MyD88. In particular, we found that miR-148 is related to *V. harveyi* infection which a typical pathogen for a wide range of marine fish, and miR-148 as a negative regulator is involved in the regulation mechanism for avoiding excessive inflammation.

miR-148 is a member of miR-148/152 family which has been indicated to involved in genesis and development of disease. Recently, miR-148/152 has been indicated to play vital roles in impairing innate response and antigen presentation of TLR-triggered dendritic cells. Up-regulated miR-148/152 has been found to inhibit the production of cytokines on dendritic cells by targeting CaMKIIa^26^. However, the regulation mechanism by which miR-148/152 modulates the immune response in fish is rare to explore. In this paper, we demonstrated that overexpression of miR-148 significantly attenuated the production of inflammatory cytokines through directly targeting MyD88. Importantly, overexpression of miR-148 also inhibited the activation of NF-κB signaling.

In summary, we demonstrate that as a negatively regulator of MyD88, miR-148 could be upregulated by *V. harveyi*, as well as LPS. Moreover, miR-148 suppresses NF-κB signaling and the production of inflammatory cytokines, including IL-1β, IL-6 after LPS stimulation. All these results not only suggest miR-148 as a new negative regulator involved MyD88-meidated immune response but also indicate a novel mechanism for avoiding excessive inflammation in teleost fish, as well as in mammals.

## Material and Methods

### Animals and challenge

Miiuy croakers (average weight, 750 g) were obtained from Zhoushan Fisheries Research Institute (Zhejiang, China). For the stimulation experiment, briefly, these healthy fishes were randomly divided into two groups in which the experimental individuals were challenged with 1 ml *V. harveyi* (1.5 × 10^8^ CFU/ml) or 1 ml suspension of LPS (1 mg/ml) through intraperitoneal and the other individuals kept in separate tanks were corresponding challenged with 1 ml physiological water as the control. Fishes were respectively killed in various times and tissues were collected and then stored at −80 °C for later use. All animal experiments were performed in accordance with the National Institutes of Health’s Guide for the Care and Use of Laboratory Animals, and the experimental protocols were approved by the Research Ethics Committee of the College of Marine science, Zhejiang Ocean University (No. EC2015011).

### Macrophages culture and stimulation

Miiuy croaker was killed and then swabbed with 75% alcohol. Head-kidney was removed aseptically and placed in ice-cold L-15 cell culture. After washing, the sample was minced thoroughly with scissors. Subsequently, the sample were passed through a 100 um nylon mesh in L-15 medium containing penicillin (100 IU/ml), streptomycin (100 µg/ml), 2% foetal bovine serum (FBS) and heparin (20 U/ml). The suspension was placed on a 34%/51% Percoll (Pharmacia, USA) gradient and centrifuged at 400 g for 40 min at 4 °C ^27^. Whereafter, cells at the interfaces were collected and washed twice in L-15 medium. Cells were counted in a haemocytometer and their viability was determined by the trypan blue (Sigma, USA). Cell population profiles obtained from the Percoll gradient interfaces were analyzed by Wright’s-Giemsa staining.

For the LPS exposure, macrophages were cultured in plates at a density of 4 × 10^7^ cells per well in L-15. The next day, the cell pellet was resuspended in fresh complete L-15 medium supplemented with 20% FBS containing 10 µg/ml of lipopolysaccharides (Sigma, USA), and physiological water was used as stimulated controls. The macrophages were harvested in different times for RNA extraction. Cells with PBS stimulation were collected as the control, and each experiment had three biological replicates.

### RNA isolation and real-time quantitative PCR

Total RNA and sRNA (<200 nt) were isolated with TRIzol reagent (Invitrogen) and miRcute miRNA Isolation Kit (Tiangen), respectively, following the manufacturer’s protocol. Real-time quantitative PCR was performed on a 7300 real-time PCR system (Applied Biosystems, USA) as we previously described^27^. The relative expression of mRNA was normalized to that of internal control β-actin. And the relative expressions of miRNAs were normalized to that of internal control 5.8 s rRNA. All the amplification reactions were carried out within triplicate well of each sample and sequences of mRNA and miRNA primers were lists in Supplemental Table 1.

### Plasmid construction and Transfection

To construct the MyD88 expression vector, the full length CDS region and 3′UTR of miiuy croaker MyD88 gene were amplified by specific primer pairs with Flag tag and restricted endonuclease sites *Kpn* I and *EcoR* I, and then inserted into pcDNA3.1 vector (Invitrogen). To construct MyD88-3′UTR reporter vector, the full length 3′-UTR region of MyD88 was amplified from cDNA of miiuy croaker. The PCR product was digested within *Nhe* I and *Sal* I, respectively, which was then cloned into pmirGLO luciferase reporter vector (Promega). The mutant-type of MyD88-3′UTR reporter vector were conducted using Mut Express II Fast Mutagenesis Kit V2 (Vazyme) with specific primers (Supplemental Table 1). Additionally, MyD88-3′UTR or mutant-type was cloned into the pIZ/V5-His vector (Invitrogen) which contained the sequence of enhanced green fluorescent protein (GFP). To construct pre-miRNA vector, the pre-miR-148 sequence were amplified by PCR and then cloned into pcDNA6.2-GW/EmGFP vector (Invitrogen). All the recombinant plasmids were extracted through Endotoxin-Free Plasmid DNA Miniprep Kit (Tiangen) and confirmed by Sanger sequencing before the dual-luciferase reporter assay. Before transient transfection, HEK293 cells were seeded in 24-well plates for 24 h. Cells were subsequently transfected with 100 ng plasmids using Lipofectamine 2000^TM^ (Invitrogen), according to the manufacturer’s instructions.

### miRNA mimics and inhibitors

miR-148 mimics (dsRNA oligonucleotides), miR-148 inhibitor (single-stranded oligonucleotides chemically modified by 2′-Ome) and control oligonucleotides were commercially synthesized by GenePharma (Shanghai, China). Nonspecific RNA mimics (mimics control) or inhibitors (inhibitors control) were transfected as negative controls, and they have similar chemical properties as the miR-148 mimics or miR-148 inhibitor, respectively. Their sequences are as follows: miR-148 mimics, sense: 5′-UCAGUGCAUUACAGAACUUUG-3′, antisense: 5′-AAGUUCUGUAAUGCACUGAUU-3′; mimics control, sense: 5′-UUCUCCGAACGUGUCACGUTT-3′, antisense: 5′-ACGUGACACGUUCGGAGAATT-3′; miR-148 inhibitor, 5′-CAAAGUUCUGUAAUGCACUGA-3′; and inhibitors control, 5′-CAGUACUUUUGUGUAGUACAA-3′. HEK293 cells or macrophages were transfected with 30–100 nM of each oligonucleotides 24 h before LPS stimulation using Lipofectamine 2000^TM^ (Invitrogen).

### RNA interference

The MyD88-specific siRNA were 5′-GCUCGAAACAAACGCCUUATT′3′ (sense) and 5′-UAAGGCGUUUGUUUCGAGCTT-3′ (antisense). The scrambled control RNA sequences were 5′-UUCUCCGAACGUGUCACGUTT-3′ (sense) and 5′-ACGUGACACGUUCGGAGAATT-3′ (antisense). The MyD88-specific siRNA transfection was performed with Lipofectamine 2000^TM^ (Invitrogen). Macrophages were transfected with 100 nM of each siRNA for 24 h before LPS stimulation.

### Dual-luciferase reporter assays

For miRNA target identification, HEK293 cells were cotransfected with wild type or mutant type MyD88-3′UTR luciferase reporter vector, along with miR-148 mimics, inhibitors and controls or pre-miR-148 plasmid. Additionally, HEK293 cells were cotransfected with NF-κB, ISRE luciferase reporter plasmid, pRL-TK Renilla luciferase plasmid, MyD88 expression plasmid, along with either miR-148 mimics, inhibitors and controls or pre-miR-148 plasmids for dual-luciferase report assay. After 24 h or 48 h, the cells were collected for reporter activity using the Dual-Luciferase Reporter System (Promega) and the relative luciferase activity value was achieved against the renilla luciferase control. The results shown were done in triplicate for each experiment, and three independent experiments were conducted.

### Western blotting

After 48 h post-transfection, total HEK293 cellular lysates or macrophages lysates were generated using 1 × SDS-PAGE loading buffer, respectively. Then SDS-PAGE was performed, followed by protein transfer to PVDF membranes (Pall Corporation) using semi-dry (Bio-Rad Trans Blot Turbo System). Membranes were incubated at 4 °C overnight with anti-Flag mouse monoclonal antibody (Beyotime) and polyclonal anti-MyD88 antiserum^16^ or GAPDH monoclonal antibodies, respectively. The following day, the membranes were incubated with the secondary antibody conjugated with horseradish peroxidase at room temperature for 60 min (Beyotime). The immunoreactive proteins were detected by using BeyoECL Plus (Beyotime) and digital imaging was performed with a cold CCD camera.

### Statistical analysis

Data on relative gene expression was obtained using the 2^−∆∆CT^ method, and comparisons between groups were analyzed by one-way analysis of variance (ANOVA) followed by Duncan’s multiple comparison tests^28^. All data were presented as the mean ± SE, significant differences between groups were determined by two-tailed Student’s t-test.

## Electronic supplementary material

Supplemental information
