# Supplementary material for: MicroRNA-148 as a negative regulator of the common TLR adaptor mediates inflammatory response in teleost fish
Source: Sci Rep. 2017 Jun 23;7:4124. doi: 10.1038/s41598-017-04354-9 (PMC5482802; doi:10.1038/s41598-017-04354-9)
Supplement: Supplementary file 1 — Supplemental information [file 41598_2017_4354_MOESM1_ESM.pdf]

Supplemental Information

MicroRNA-148 as a negative regulator for the common TLR adaptor mediated inflammatory response in teleost fish

Qing Chu#, Yunhang Gao#, Dekun Bi, Tianjun Xu*

Laboratory of Fish Biogenetics & Immune Evolution, College of Marine Science, Zhejiang Ocean University, Zhoushan, 316022, China

# Contributed equally.

*Corresponding author. Dr. Tianjun Xu

E-mail: tianjunxu@163.com

Running title: microRNA-148 and Myd88 modulate NF-κB pathway

Supplemental Figure 1

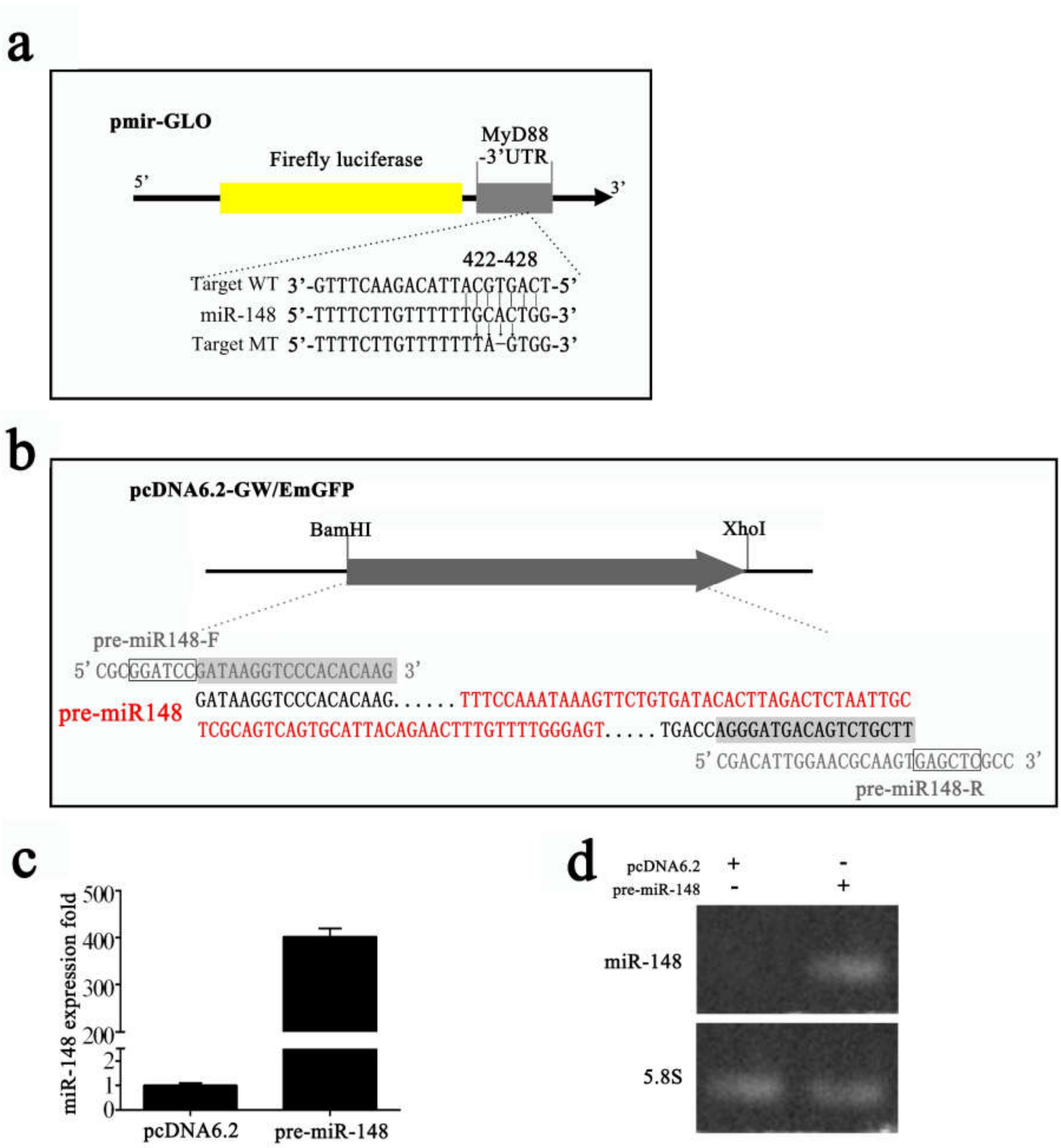

Supplemental Figure 2

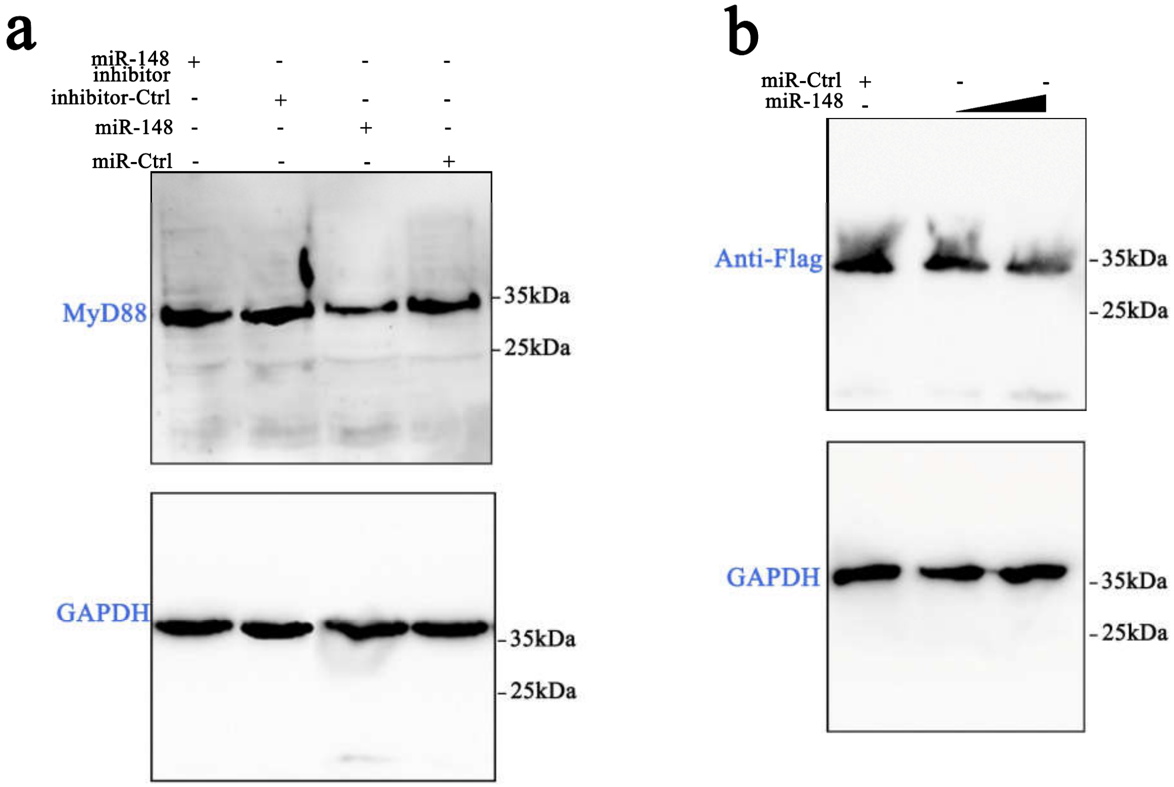

Supplemental Table 1

Primers	Sequences (5'-3')
**Real-time PCR**	
miR-148-qRT-F	GCAGTCAGTGCATTACAGA
miR-148-qRT-F	TCCAGTTTTTTTTTTTTTTTCAAAGTTC
5.8S rRNA-qRT-F	AACTCTTAGCGGTGGATCA
5.8S rRNA-qRT-R	GTTTTTTTTTTTTTTTGCCGAGTG
MyD88-qRT-F	AGTTGGAACAGACCGAGTA
MyD88-qRT-R	TGAGGAAGCGTAAGATGC
IL6-qRT-F	GCGGTAAAGGCATGGATAT
IL6-qRT-R	GTTGTAGTTGGAAGGGCAG
IL1β-qRT-F	CATAAGGATGGGGACAACGAG
IL1β-qRT-R	TAGGGGACGGACACAAGGGTA
β-actin-qRT-F	GAGCCGCACGCTTCTTT
β-actin-qRT-R	CTGCTGTAGCCGAGGAC
**Vector construction**	
pre-miR-148-F	CGCGGATCCGATAAGGTCCCACACAAG
pre-miR-148-R	CCGCTCGAGTGAACGCAAGGTTACAGC
MyD88-3'UTR-WT-F	CTAGCTAGCCATTGCCCAAACATTACAGG
MyD88-3'UTR-WT-R	ACGCGTCGACCGAGGGATGCACATTCCGAT
MyD88-3'UTR-MT-F	TTTTGCAACGCCACTTCCTCTTGTGAGTCAACT
MyD88-3'UTR-MT-R	GGAAGTGGCGTTGCAAAAAACAAGAAAAAAAAAACA
MyD88-F	CGGGGTACCATGGCGTGTTGCGATAAATC
MyD88-R	CCGGAATTCGAGGGATGCACATTCCGATA
GFP-MyD88-3'UTR-F	CCCAAGCTTGCTAGCATTGCCCAAACATTACAGG
GFP-MyD88-3'UTR-R	CGCGGATCCCGAGGGATGCACATTCCGAT

Supplemental Figure Legends

Supplemental Figure 1. (a) Sequence alignment of miR-148 and the construction of luciferase report plasmids. (b) Sequence alignment of pre-miR-148 and its construction plasmids. (c) Pre-miR-148 and pcDNA6.2 plasmids was transfected into HEK293 cells. 24 h post-transfection, qPCR was conducted to detect miR-148. (d) 24 h post-transfection, RT-PCR was conducted to detect miR-148.

Supplemental Figure 2. Un-cropped blots from main figures. (a) MyD88 and GAPDH blots from Western blot from Fig. 4a. (b) Flag and GAPDH blots from Western blot from Fig. 4c.

Supplemental Table 1. PCR primer information in this study.
